# DIFFERENCES IN HOSPICE AND NONHOSPICE NURSES’ PERSONAL AND PROFESSIONAL CHARACTERISTICS

**DOI:** 10.1093/geroni/igad104.2581

**Published:** 2023-12-21

**Authors:** Janie Taylor, Renee’ Zucchero

**Affiliations:** Xavier University, Cincinnati, Ohio, United States; Xavier University, Cincinnati, Ohio, United States

## Abstract

The overextension of nurses due to an aging population, increase in chronic conditions, and the COVID-19 pandemic, places them at risk of negative outcomes. However, less is known about how hospice nurses compare to nurses in other subfields in terms of professional quality of life or factors that may potentially protect against adverse consequences of caregiving. Therefore, this study investigated differences between hospice and non-hospice nurses across several factors, including the work environment, self-awareness (SA), psychological flexibility (PF), palliative care self-efficacy (SE), and three components of professional quality of life – compassion satisfaction (CS), burnout, and secondary traumatic stress (STS). Using a cross-sectional, exploratory, and quantitative design, participants were sampled through snowball and convenience techniques. Participants (N= 72) completed an online survey, 31 (43%) identifying as working in hospice and 41 (57%) as working in non-hospice settings. Independent samples t-tests were conducted. Hospice nurses reported significantly healthier work environments, higher levels of SA, SE, and CS, and lower levels of burnout than non-hospice nurses. However, there was no significant difference in PF or STS. These findings offer a novel comparison between hospice and non-hospice nurses that could inform workplace change to better support nurses as they face increased demands and increased risk for negative outcome.

